# Phosphoproteomics Identifies Significant Biomarkers Associated with the Proliferation and Metastasis of Prostate Cancer

**DOI:** 10.3390/toxins13080554

**Published:** 2021-08-09

**Authors:** Rongfang Xu, Yan Chen, Zijun Wang, Changxin Zhang, Xiaoping Dong, Yujie Yan, Ying Wang, Yong Zeng, Ping Chen

**Affiliations:** 1 The National & Local Joint Engineering Laboratory of Animal Peptide Drug Development, College of Life Science, Hunan Normal University, Changsha 410081, China; xurongfang1996@outlook.com (R.X.); cchenyann@outlook.com (Y.C.); sheueyz9@163.com (Z.W.); nongmeidashu21@gmail.com (C.Z.); dxp121110@hunnu.edu.cn (X.D.); yanyujie@hunnu.edu.cn (Y.Y.); wangying91@hunnu.edu.cn (Y.W.); 2 State Key Laboratory of Developmental Biology of Freshwater Fish, College of Life Science, Hunan Normal University, Changsha 410081, China

**Keywords:** HNTX-III, JZTX-I, prostate cancer, quantitative phosphoproteomics, bioinformatics

## Abstract

The spider peptide toxins HNTX-III and JZTX-I are a specific inhibitor and activator of TTX-S VGSCs, respectively. They play important roles in regulating MAT-LyLu cell metastasis in prostate cancer. In order to identify key biomarkers involved in the regulation of MAT-LyLu cell metastasis, iTRAQ-based quantitative phosphoproteomics analysis was performed on cells treated with HNTX-III, JZTX-I and blank. A total of 554 unique phosphorylated proteins and 1779 distinct phosphorylated proteins were identified, while 55 and 36 phosphorylated proteins were identified as differentially expressed proteins in HNTX-III and JZTX-I treated groups compared with control groups. Multiple bioinformatics analysis based on quantitative phosphoproteomics data suggested that the differentially expressed phosphorylated proteins and peptides were significantly associated with the migration and invasion of prostate tumors. Specifically, the toxins HNTX-III and JZTX-I have opposite effects on tumor formation and metastasis by regulating the expression and phosphorylation level of causal proteins. Herein, we highlighted three key proteins EEF2, U2AF2 and FLNC which were down-regulated in HNTX-III treated cells and up-regulated in JZTX-I treated cells. They played significant roles in cancer related physiological and pathological processes. The differentially expressed phosphorylated proteins identified in this study may serve as potential biomarkers for precision medicine for prostate cancer in the near future.

## 1. Introduction

Prostate cancer is one of the most common non-cutaneous genital malignancies in men, with an estimated 1.6 million cases and 366,000 deaths worldwide every year [1]. Metastatic disease is the leading cause of prostate-related death [2]. Metastatic prostate cancer resists hormone therapy and other conventional treatments [3,4]. However, high metastasis is one of the inevitable characteristics of most cancers, therefore, it is necessary and urgent to develop efficient targeted drugs to inhibit the high metastasis of prostate cancer. In the past few years, research on the role of ion channels in cancer have attached importance to the involvement of various channel types in cancer cell metabolism and the tumor microenvironment [5]. 

Voltage-gated sodium channels (VGSCs) are a kind of transmembrane glycoprotein which consist of pore-forming α-subunits (260 kDa) and auxiliary β subunits (33–36 kDa) [6]. It is known that nine different genes (SCN1A-SCN11A) encode nine distinct sodium channels (Nav1.1–Nav1.9) [7,8]. In pharmacology, according to the sensitivity of sodium channel subtypes to tetrodotoxin (TTX), sodium channels can be divided into TTX-sensitive (TTX-S) and TTX-insensitive (TTX-R) [8]. In recent years, studies in rat prostate cancer models have shown that VGSCs are related to invasive metastasis in vitro [9], and TTX-S VGSCs can reduce the invasiveness of prostate cancer cells [10,11,12], which indicates that VGSCs may play an important role in tumor migration. Hainan Toxin-III (HNTX-III) is a 33 amino acid peptide toxin isolated from the venom of the Chinese bird spider (*Ornithoctonus hainana* that can reduce the activity of TTX-S VGSCs [13]. Jingzhao Toxin-I (JZTX-I) is a 33 amino acid and three disulfide bonds peptide toxin from the venom of the spider *Chilobrachys jingzhao* [14]. It can delay the rapid inactivation mechanics of TTX-S VGSCs [13]. The effect of the spider peptide toxins HNTX-III and JZTX-I on MAT-LyLu in prostate cancer cells is a very interesting and innovative study. In our previous work, the spider peptide toxins HNTX-III and JZTX-I have been used in the treatment of prostate cancer, and the preliminary cellular study suggested that both of them could regulate the metastasis of prostate cancer [15]. To better understand the underlying molecular mechanisms of the regulation, we have now performed comparative phosphoproteomics in two groups: HNTX-III treatment (114 isobaric tags for relative and absolute quantification (iTRAQ) tags) with blank group (116 iTRAQ tags) are named as experimental group I; the JZTX-I treatment (115 iTRAQ tags) with the blank group (116 iTRAQ tags) are named as experimental group II.

Protein phosphorylation has a profound effect on the dynamic processes of the cell because the role of kinases and phosphatases is the basis of many major biological functions [16]. LC-MS-based phosphoproteomics is an efficient and powerful method in recognizing large amounts of phosphorylated proteins and studying cell signaling pathways. Phosphoproteomics can reveal the key role of phosphorylated proteins in the signaling pathways. It has been widely applied in exploring the roles of multiple toxins such as *Clostridioides difficile*, *Microcystis aeruginosa* and snake venom in human diseases [17,18,19]. Multiple bioinformatics analysis based on quantitative phosphoproteomics data help us to better understand the biological function and topological associations of the significantly differentially expressed phosphorylated proteins (DEPs) between case and control groups. It may largely help us to uncover the underlying molecular mechanisms of prostate cancer regulation by the peptide toxins HNTX-III and JZTX-I.

## 2. Results

### 2.1. Identification of Phosphorylated Proteins and Phosphopeptides in the MAT-LyLu Cells

**Figure 1:**
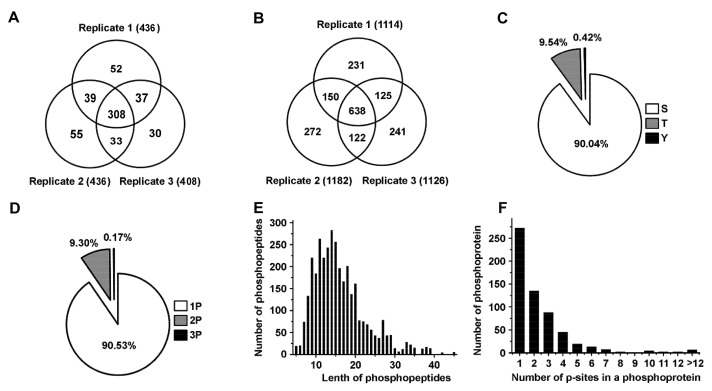
Statistical information of phosphoproteomics in the Mat-lyLu cells based on iTRAQ method. (**A**,**B**) showed the phosphoproteins and phosphopeptides identified in the triplicate analyses respectively based on iTRAQ method. (**C**) The proportions of different phosphorylation sites (serine (p-Ser), threonine (p-Thr) and tyrosine (p-Tyr)); (**D**) Distribution of phosphopeptides depending on their number of p-sites; (**E**) Distribution of phosphopeptides based on their length; (**F**) Distribution of phosphorylation proteins based on their number of p-sites.

To identify significant biomarkers involved in toxin regulation of prostate cancer, we conducted a quantitative phosphoproteomic analysis by iTRAQ integrated with LC-MS/MS to identify differentially expressed phosphorylated proteins and phosphopeptides in high metastatic MAT-LyLu cells. To obtain statistically reliable data, triplicate runs were performed for protein and phosphopeptide identification and quantification. We successfully identified 436, 436, and 408 phosphorylated proteins in replicate 1, replicate 2 and replicate 3, respectively. At the same time, 1144, 1182 and 1126 phosphorylated peptides were successfully identified in replicate 1, replicate 2 and replicate 3, respectively (Figure 1A,B, Supplementary Materials, Tables S1 and S2). In total, 554 non-redundant phosphorylated proteins and 1779 distinct phosphopeptides were identified. Among the 554 identified proteins, 308 common proteins were identified in three replicates; more than 75.45% (418) were identified in at least two replicates. Among the 1779 identified phosphopetides, 638 common peptides with same phosphorylation sites were identified in three replicates, more than 71.16% (1266) were identified at least two replicates.

Furthermore, we statistically analyzed the phosphorylation sites and the length of peptides. A total of 3452 phosphorylated peptides and 3784 p-sites were identified. Among all the p-sites, 3407 serine p-sites, 361 threonine p-sites and 16 tyrosine p-sites were identified, respectively. Thus, the percentage distribution of p-sites was Ser/Thr/Tyr = 90.04%/9.54%/0.42%, which was in accordance with the value reported previously (Ser:Thr:Tyr = 90%:9.9%:0.1%) (Figure 1C) [20]. Among the 3452 phosphorylation peptides, 90.53% (3125/3452) singly phosphorylated phosphopeptides, 9.3% (321/3452) doubly phosphorylated phosphopeptides, and 0.17% (6/3452) phosphopeptides carrying three p-sites (Figure 1D) were identified. The length of most phosphopeptides (2663/3452 or approximately 77.14%) ranged from 8 to 20 amino acids, (Figure 1E). Moreover, we detected the number of p-sites in a phosphoprotein, the results showed that the proteins contained one, two and three p-sites accounted for 45.71% (272/595), 22.69% (135/595) and 14.79% (88/595), respectively. 16.81% (100/595) of the proteins contained more than three p-sites (Figure 1F).

### 2.2. Identification of DEPs

**Figure 2:**
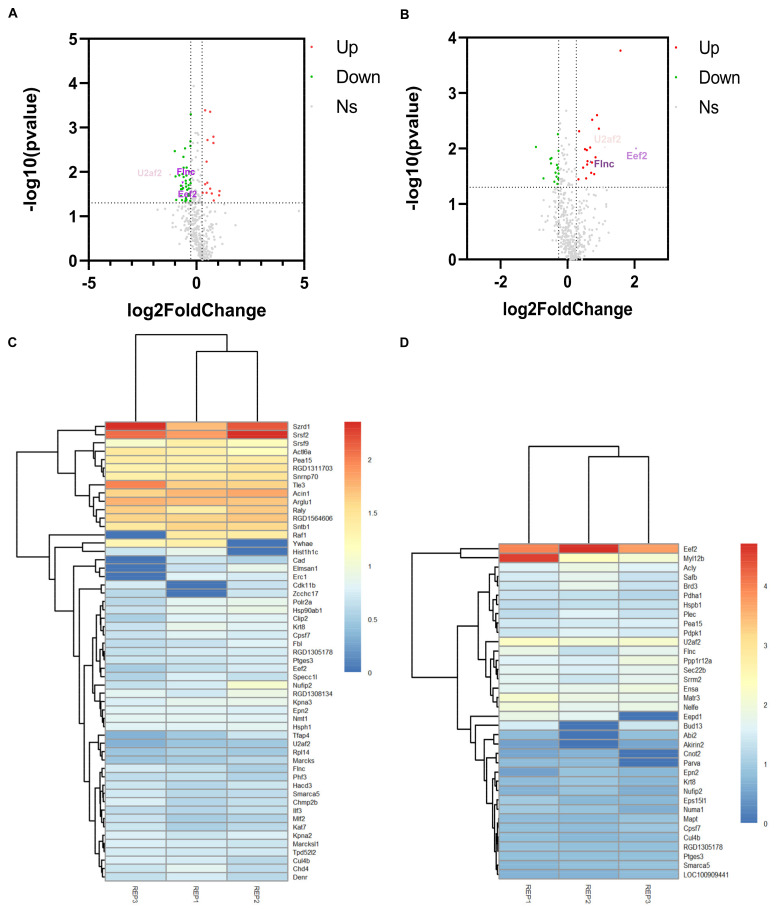
Identification of DEPs. (**A**,**B**) showed the DEPs identified in experimental group I and II, respectively. They were both compared with the control group. And each group of experiments were repeated three times. The red dots are upregulated DEPs with log2(foldchange) > 0.263. The green dots are down DEPs with log2(foldchange) < 0.263. Among them, the red triangles and the green triangles in (**A**,**B**) are the three specific DEPs that reversely regulated in experimental group I and II. (**C**,**D**) Cluster analysis based on 55 and 36 DEPs in the experimental group I and II, respectively. Red and blue show the higher and smaller fold-change of up-regulated and down-regulated DEPs, respectively. REP1: Repeat the experiment for the first time; REP2: Repeat the experiment for the second time; REP3: Repeat the experiment for the third time.

To ensure the reliability of the quantitative data, only the phosphorylated proteins (417) identified in two or more replicates were used for subsequent analysis. *p*-value < 0.05 and |log2(foldchange)| > 0.263 were set as the statistical thresholds for DEPs identification. In experimental group I (HNTX-III treatment group (114 isobaric tags for relative and absolute quantification (iTRAQ) tags) with blank group (116 iTRAQ tags)) and II (JZTX-I treatment group (115 iTRAQ tags) with the blank group (116 iTRAQ tags)), 55 (15 up-regulated and 40 down-regulated proteins) and 36 (20 up-regulated and 16 down-regulated proteins) phosphorylated DEPs were identified, respectively (Figure 2A,B). Among them, EEF2, U2AF2 and FLNC were significantly down-regulated in experimental group I and significantly up-regulated in experimental group II. The three pivotal DEPs specific statistical indicators are presented in the Table 1. The clustering of experimental group I and experimental group II in the three replicates is shown in Figure 2C,D.

### 2.3. Identification of Overrepresented Kinase Targeting Peptide Motifs

**Figure 3:**
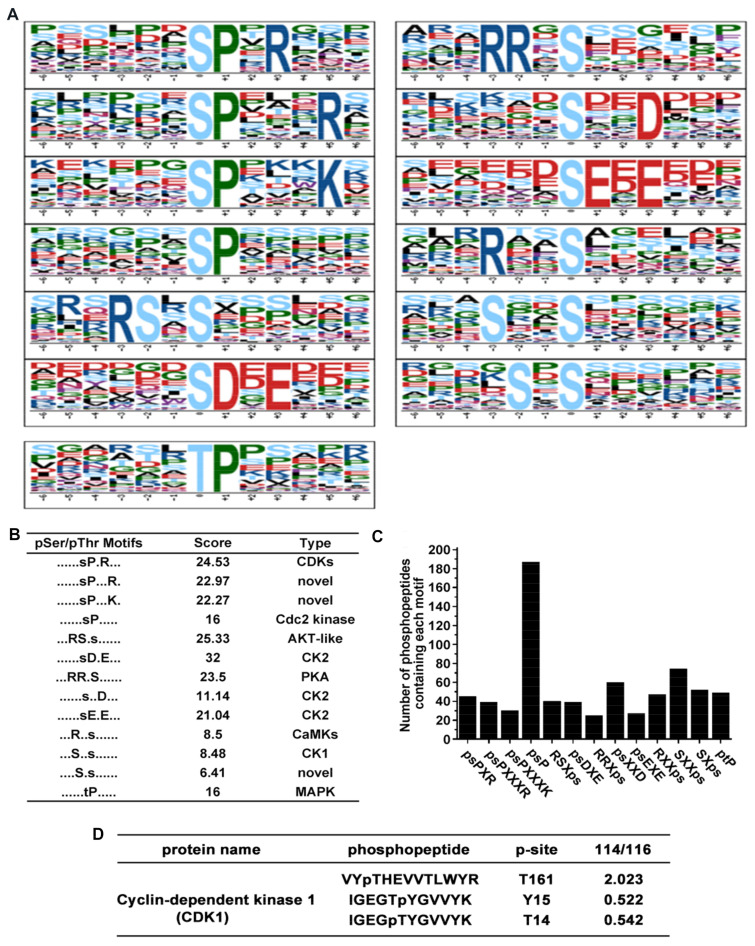
Motif analysis of identified phosphopeptides. (**A**) 12 serine motifs and one threonine motif were significantly enriched according to Motif-X online evaluation. (**B**) Score and the type of overrepresented kinase of each identified motif. (**C**) Number of phosphopeptides that contain a specific overrepresented phosphorylation motif. (**D**) Phosphorylation level of CDK1 after HNTX-III and JZTX-I treatment.

In total, 12 serine motifs and one threonine motif were significantly enriched (Figure 3A). Each motif score and their corresponding kinases are presented in Figure 3B. Among the 12 serine motifs, the top four known motifs were psDXE, RSXps, psPXR, and RRXps according to motif score, there were potential substrates of casein kinase II (CK2), AKT-like kinase, cyclin-dependent kinases (CDKs) and protein kinase A (PKA), respectively. However, ptP is the most common threonine motif which located in the nucleus, cytosol, and secreted proteins could be targeted for mitogen-activated protein kinase (MAPK) [21,22]. A column diagram showed that the number of phosphopeptides that contain each of the overrepresented phosphorylation motif, we observed a lot of phosphopeptides containing the psP motif (Figure 3C), which is a known target for proline-directed kinase including cyclin-dependent kinases and mitogen-activated kinases. Remarkably, our result shows that the phosphorylation level of cyclin dependent kinase 1 (CDK1) was significantly changed (Figure 3D).

### 2.4. Gene Functional Annotation

**Figure 4:**
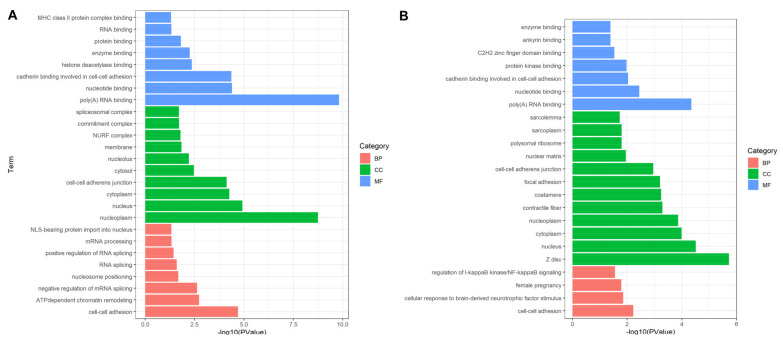
Functional annotation and enrichment analysis based on DEPs. (**A**,**B**) showed significant terms that the DEPs enriched in for experimental group I and II. Most of DEPs including EEF2, U2AF2 and FLNC from both groups are enriched in cell activity related terms such as “cell-cell adhesion”, “negative regulation of mRNA splicing, via spliceosome” and “RNA splicing”. BP: biological process; CC: cell component; MF: molecular function.

In biological processes (BP), the DEPs identified in experimental group I were mainly significantly enriched in “cell-cell adhesion”, “ATP-dependent chromatin remodeling”, “negative regulation of mRNA splicing, via spliceosome”, “nucleosome positioning”, “RNA splicing”, “positive regulation of RNA splicing”, “mRNA processing” and “NLS-bearing protein import into nucleus”. “Cell adhesion” and “spliceosome” were closely related to cell proliferation. The molecular function (MF) is mainly involved in “cell-cell adhesion” and “cadherin binding involved in cell-cell adhesion”, which easily leads to the occurrence of cancer (Figure 4A). The DEPs identified in experimental group II accounted for multiple entries, including “cell adhesion”, “actin filaments”, “phosphorylation protein kinase regulation”, they are significantly related to cancer [23]. As for molecular functions, they mainly involved in “poly(A) RNA binding”, “nucleotide binding”, “cadherin binding involved in cell-cell adhesion”, “protein kinase binding”, “C2H2 zinc finger domain binding”, “ankyrin binding” and “enzyme binding” (Figure 4B). These items play key roles in regulating the changes of cell protein phosphorylation level, participating in cell signal transduction and cell migration. Subcellular localization analysis showed that DEPs are mainly located in exogenous components of cytoplasmic membrane, the expected results supported that toxin stimulation mediated VGSCs leads to changes in membrane protein expression, resulting in cell adhesion. Among them, candidate proteins EEF2, U2AF2 and FLNC were significantly annotated in “cell adhesion” (*p*-value: 6.02 × 10^−3^). “Cell adhesion”, as the most prominently annotated cell process, is closely related to the occurrence of cancer. Once the proteins involved in this process are overexpressed, it is very easy to cause tumors [24,25,26].

### 2.5. DEPs Based Pathway Analysis

**Figure 5:**
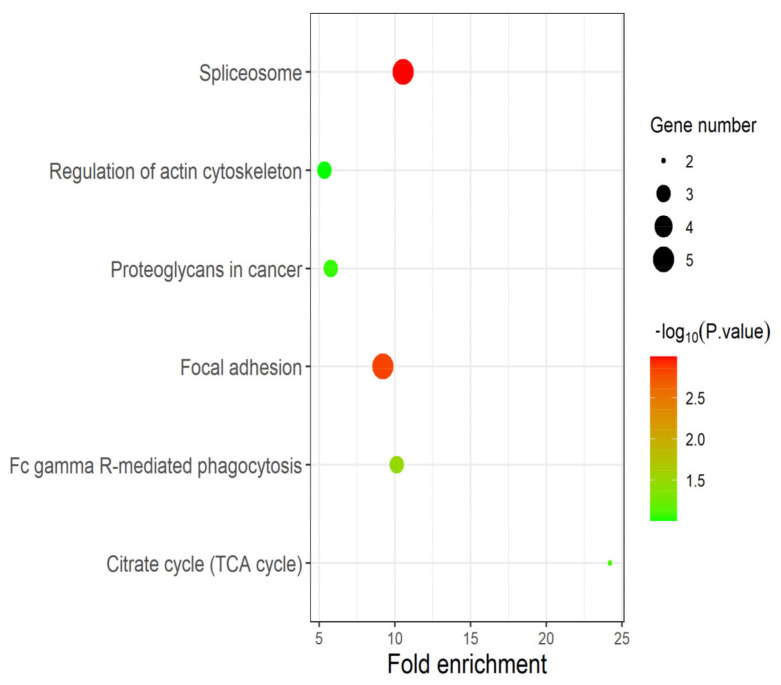
Pathway analysis based on DEPs. The results of pathway enrichment analysis based on DEPs identified in experimental group I and II. As expected, the candidate proteins EEF2, U2AF2 and FLNC play multiple and crucial roles in cancer-related pathways such as “focal adhesion”, “spliceosome” and “TCA cycle”.

The DEPs identified in experimental group I and II were significantly enriched in the “spliceosome”, “regulation of actin cytoskeleton”, “proteoglycans in cancer”, “focal adhesion”, “citrate cycle” (TCA cycle) and “FC gamma R-mediated phagocytosis” pathways (Figure 5). Among them, candidate proteins EEF2, U2AF2 and FLNC are significantly enriched in the three pathways of “spliceosome” (*p*-value: 1.01 × 10^−3^), “proteoglycans in cancer” (*p*-value: 8.58 × 10^−2^) and “focal adhesion” (*p*-value: 1.48 × 10^−3^). “Spliceosome” and “Fc gamma R-mediated phagocytosis” pathways were closely associated with the proliferation and metastasis of prostate cancer cells. “Focal adhesion” may lead to uncontrolled proliferation of cells, which is one of the main factors of tumor formation. “TCA cycle” is the central pathway for oxidative phosphorylation of cells and meets their biological energy, biosynthesis and redox balance requirements [27]. With the formation and development of tumors, changes in cell and enzyme activity may alter the composition and structure of proteoglycans, and thus alter their function. Many cancers, including prostate cancer, use these proteoglycan changes to promote their survival, growth, and spread [28].

### 2.6. The Construction of PPI Network

**Figure 6:**
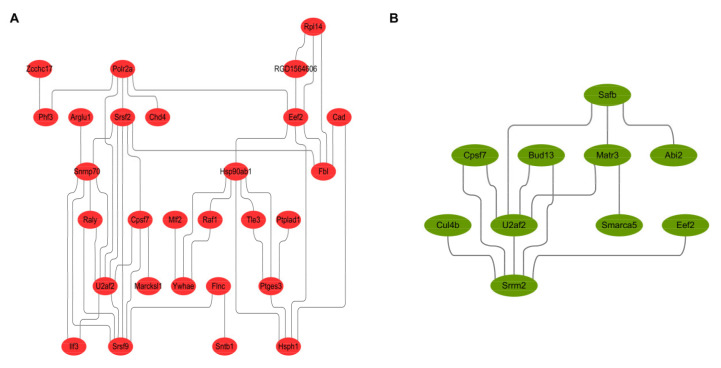
Protein-protein interaction analysis and network reconstruction based on differentially expressed genes. (**A**,**B**) showed the protein-protein interactions of DEPs from experimental group I and II. The results indicated that the candidate proteins EEF2, U2AF2 and FLNC could regulate the proliferation and metastasis of prostate cancer cells in a coordinated way with other DEPs or their upstream and downstream key factors.

The protein-protein interaction network diagram constructed by the DEPs identified in experimental group I (Figure 6A) and experimental group II (Figure 6B) shows that candidate proteins EEF2, U2AF2 and FLNC are key proteins in the PPI network. They can coordinate with other DEPs or their upstream and downstream key factors to regulate the proliferation and metastasis of prostate cancer cells.

### 2.7. Analysis of Gene-Disease Associations

**Figure 7:**
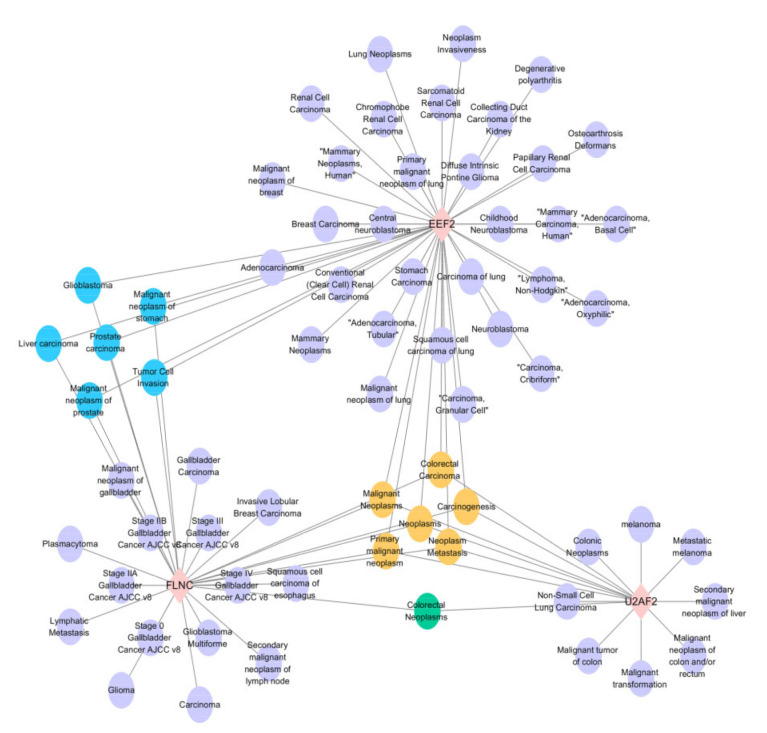
Gene-disease association study of the 3 candidate factors. The results indicated that the candidate proteins EEF2, U2AF2 and FLNC mainly involved in the diseases of “malignant neoplasms”, “carcinogenesis” and “neoplasia”.

In order to focus on understanding the diseases associated with the DEPs identified in experimental group I and experimental group II, the gene-disease association was analyzed. Among them, we mainly focused on the disease types associated with three key factors: EEF2, U2AF2 and FLNC, which were significantly down-regulated in experimental group I and significantly up-regulated in experimental group II. According to the network diagram (Figure 7), it can be seen that these three types are involved in the formation of various diseases, among which, they are all related to malignant neoplasms, neoplasms, primary malignant neoplasm, carcinogenesis and neoplasia. Both EEF2 and FLNC were associated with the formation of prostate cancer.

## 3. Discussion

In this study, we aimed to elucidate the biological activity of HNTX-III and JZTX-I toxins on migration, invasion and proliferation of MAT-LyLu cell lines in prostate cancer. Tumor metastasis and invasion are multistep and extremely complex biological processes including the detachment, angiogenesis, colonization and proliferation which could be regulated by various signaling pathways [29]. To intuitively understand the effects of these two toxins, we used the iTRAQ method to conduct quantitative phosphorylated proteomics analysis to compare the changes of protein expression level between case (HNTX-III and JZTX-I treated MAT-LyLu cells) and control samples. Quantitative phosphoproteomics integrated with multiple bioinformatics analysis revealed a series of phosphopeptides and candidate proteins such as EEF2, U2AF2 and FLNC involved in tumor metastasis and invasion. It can be clearly concluded that the three differentially phosphorylated proteins, EEF2, U2AF2 and FLNC, were down-regulated in experimental group I and up-regulated in experimental group II. They play significant roles in cancer-related pathways including “cell adhesion”, “spliceosome” and “cadherin binding involved in cell-cell adhesion” by forming synergistically functional regulatory networks. The results may largely help us to uncover the potential molecular mechanisms of HNTX-III and JZTX-I in regulating tumor metastasis and invasion.

In most tumors, the signaling pathways driven by protein kinases are significantly altered. By motif analysis of peptide motifs, the first four motifs in the 12 serine motifs were psDXE, RSXps, psPXR, and RRXps according to their motif scores, and their corresponding kinases were CK2, AKT kinase, CDKS, and PKA [21]. Among them, P21 activated kinase, a serine/threonine kinase family, is involved in a variety of tumor-related signaling pathways as a downstream node, including regulating cytoskeleton remodeling and cell movement, affecting cell proliferation and regulating apoptosis [30]. The basis of the structural homology of PAK subtype can be divided into two groups: the first group PAKs including PAK1–3 and the second group PAKs including PAK4–6 [31]. A large number of literature references have shown that PAK2 and PAK4 are directly involved in the regulation of tumor invasiveness and metastasis by influencing their client protein behaviors. In addition, our study found significant changes in other kinases. CDK1 plays an important role in mammalian cell cycles by regulating the G2/M phase transition of eukaryotic mitosis, and its abnormal expression has been reported to be associated with the proliferation and survival of breast cancer and epithelial ovarian cancer [32,33,34]. Higher CDK1 activity is associated with several apoptotic conditions, such as HIV-1 induced apoptosis [35,36]. Recently, it has been reported that protopine, as a microtubule stabilizing agent, regulates mitochondria-mediated signaling pathways by increasing CDK1 activity, including the regulation of phosphorylation of Bcl-2 and McLl-1down, so as to promote apoptosis in hormone-refractory prostate cancer [37]. It has also been reported that microtubule interferers (MIAs) lead to mitosis blockage and subsequent apoptosis by continuously activating CDK1, and CDK1 acts as a switch to control apoptosis block [38]. Therefore, our next research focus is to determine that HNTX-III may be a novel microtubule stabilizer that inhibits tumor metastasis and induces apoptosis by significantly enhancing CDK1 activity, and to try to explore its mechanisms.

Eukaryotic extension factor 2 (EEF2) plays important roles in the GTP-dependent translocation of ribosomes along mRNA and proliferation, migration and survival of various cancers. Various experiments have proved that EEF2 may be the direct target regulated by *Mir-183-5P* in gastric carcinoma (GC) [39]. The interaction between Hspa5 and EEF2 promotes the overexpression of PRMT7 in non-small cell lung cancer cells, thus regulating cancer cell migration and colony formation [40]. In addition, EEF2 gene products have immunogenicity and are expected to be target molecules for a variety of cancer immunotherapies [41]. It has been shown that IHC-detected EEF2 expression is significantly correlated with higher prostate-specific antigen, Gleason score and TNM stage. Therefore, it can be used as a potential biomarker for the evaluation of prostate [42]. However, EEF2, was annotated as “cell adhesion” and “cadherin binding involved in cell-cell adhesion” under the action of both toxins. Cell adhesion is the attachment of one cell to another cell by attachment molecules. Once the proteins involved in this process are overexpressed, they are highly likely to cause the occurrence of cancer [22]. Tumor is a complex tissue in which constituent cells are held together by forces generated by cell-cell and cell-ECM interactions. The direct cell-cell adhesion is usually largely ascribed to the hydrophilic interaction between cadherin [43].

U2 snRNP auxiliary factor large subunit (U2AF2) is one of the earliest events of the spliceosome and plays a key role in the spliceosome signaling pathway. Filamin C (FLNC) works as a large actin-cross-linking protein in various cells. According to the literature, the transient expression or silencing of FLNC can affect the proliferation and colony formation of cancer cells and silencing of endogenous FLNC can enhance the migration and invasion of cancer cells [44]. “Spliceosome”, “tumorigenesis”, “focal adhesion” and “TCA cycle” were enriched in these two toxins, respectively. It has been reported that the overexpression of MYC gene is one of the most common driving factors of human cancer [45,46], and the spliceosome is a new target of oncogenic stress in MYC-driven cancer [47], so the spliceosome play a significant role in cancer physiology and pathology by mediating gene drive. Among them, the phosphorylated protein U2AF2 is a key protein in the signaling pathway of the spliceosome, and the important functional surface of its splice factor is also related to cancer-related mutations [48]. Compared with normal cells, the partial inhibition of spliceosome prevents the removal of introns in cancer cells, the maturation of pre-mRNA and many necessary cell processes, thus interfering with the division, proliferation and maturation of cancer cells and inhibiting cancer cells [49]. Cell adhesion kinase (FAK) is a kind of tyrosine kinase, and cytoplasm mediated kinase signaling pathways plays a very important role in humans in the amplification, expression and activation of a variety of malignant tumors [50]. Studies have shown that the FAK signaling pathway is involved in cell proliferation, migration, angiogenesis, invasion, survival and epithelial-interstitial transformation through multiple pathways [51]. In addition, FAK appears to play a role in tumor metabolism, promoting glucose consumption and fat generation, and promoting cancer cell proliferation, migration and survival [52]. Some cancer cells, especially those with uncontrolled expression of oncogenes and suppressive genes, rely on the TCA cycle for energy production and macromolecule synthesis [53]. Targeted reprogramming of metabolic pathways, including the TCA, could provide a new and promising therapeutic pathway for a wide range of cancer treatments [27,54].

## 4. Conclusions

HNTX-III and JZTX-I have significant effects on the highly metastatic prostate cancer cell lines MAT-LyLu. HNTX-III can inhibit tumor formation, while the toxin JZTX-I can promote the proliferation and migration of cancer cells. In this paper, phosphoproteomics integrated with bioinformatics analysis showed that the above two toxins can regulate the proliferation and metastatic of cancer cells through mediating the expression level of significant biomarkers such as EEF2, U2AF2 and FLNC. They showed reverse expression trend in the experimental group I (significantly downregulated) and group II (significantly upregulated). Further biological function analysis showed that EEF2, U2AF2 and FLNC are related to the molecular functions of tumor cell proliferation, metastasis and spreading, “cell adhesion”, “cadherin binding and participation in cell-cell adhesion”, and are significantly enriched in signal pathways closely related to cell proliferation, migration, angiogenesis, invasion, survival, and epithelial-mesenchymal transition, such as “splicesome”, “tumorogenesis”, “focal adhesion” and “tricarboxylic acid cycle”. Previous studies have shown that HNTX-III and JZTX-I can reduce and enhance the activity of TTX-sensitive (TTX-R) voltage-gated sodium channels (VGSCs), respectively. However, the main purpose in this study was to investigate the variation of protein regulation and phosphorylation in the prostate cancer cell lines MAT-LyLu and further expected to decipher the potential molecular mechanisms under different experimental conditions. 55 and 36 were identified as significant DEPs in the prostate cancer cell lines MAT-LyLu treated by HNTX-III and JZTX-I respectively. Multiple bioinformatics analysis suggested most of DEPs are enriched the terms “tricarboxylic acid cycle” and “regulation of actin cytoskeleton” or the pathway “proteoglycans synthesis” and “focal adhesion” and “splicesome”, they may either participate in the processes of energy providing for cell survival and movement or play significant roles in cell invasion, adhesion and migration. Therefore, we speculate that TTX-S type VGSCs modulators HNTX-III and JZTX-I can affect the proliferation, metastasis and invasion of prostate cancer cells by regulating the expression and phosphorylated level of causal proteins (potential biomarkers) such as EEF2, U2AF2 and FLNC and the related processes including “energy metabolism and transition” and “cell adhesion and migration”. The results in this study may server as a significant reference map for further functional study focused on single significant biomarker. Taken together, this study identified several key proteins that associated with prostate cancer proliferation and metastatic. The results may help us to better understand the potential molecular mechanisms of toxin regulation in specific cancer, meanwhile suggest possible biomarkers or targets for prostate cancer therapy.

## 5. Materials and Methods

### 5.1. Cell Culture and Treatment

Rat prostate cancer MAT-LyLu cell lines was purchased from American Type Culture Collection (Rockville, MD, USA). Dulbecco’s modified Eagle’s (DMEM) medium, fetal bovine serum (FBS), L-glutamine and antibiotics were all purchased from Invitrogen (Carlsbad, CA, USA). Spider peptide toxins HNTX-III and JZTX-I were separated and purified in our laboratory [13,14]. Rat prostate cancer MAT-LyLu cell lines were cultured in DMEM medium supplemented with 10% (FBS), antibiotic-antimycotic mixture (100 IU/mL penicillin, 100 μg/mL streptomycin) and 2 mM L-glutamine. Cells were incubated with 5% CO_2_ at 37 °C, and 95% humidity. When the cell density reached 80–90%, 5 μM spider peptide toxin HNTX-III and 5 μM spider peptide toxin JZTX-I were added to the two experimental groups and incubated for 24 h, respectively. The control group was treated with equal amounts of fresh growth medium and incubated for the same time.

### 5.2. Protein Extraction, Digestion and iTRAQ Labeling

There were three main steps for sample preparation in this study. Firstly, the cells were washed with cold phosphate buffered saline and lysed in lysis buffer containing 6 M urea, 0.1 M Tris-HCl (pH = 8.5). The supernatant after centrifugation at 14,000× *g* for 15 min was collected, the protein concentration was determined by BCA protein assay. Secondly, DL-dithiothreitol (DTT) with a final concentration of 5 mM was added to 1 mg protein and incubated at 37 °C for 2 h reduction reactions, iodoacetamide (IAA) at a final concentration of 5 mM was added and incubated at room temperature for 30 min alkylation reaction in darkness. The samples were diluted with 2 M urea in 0.1 M Tris-HCl (pH 8.0) and 25 mM NH_4_HCO_3_, and then, a digestion process was conducted with trypsin at 37 °C for 14 h. Thirdly, the tryptic peptides were desalted using a C18 reverse phase column (Waters, Milford, MA, USA) and eluted peptides were lyophilized and subjected to phosphopeptide enrichment. Protein extracts from HNTX-III and JZTX-I treated cells were labeled with 114, 115 iTRAQ tags, respectively, while those of the control were labeled with 116 iTRAQ tags. Then the two iTRAQ labeled samples were mixed into one sample by 114 and 116, 115 and 116, and lyophilized for chromatographic fractionation.

### 5.3. Phosphopeptide Enrichment

Tryptic peptides were fractionated by strong cation exchange fractionation chromatography. Briefly, labeled peptides were redissolved in mobile phase A (98% H_2_O and 2% acetontrile (ACN), pH = 10) and mobile phase B (98% ACN and 2% H_2_O, pH = 10), elution was carried out at a flow rate of 0.7 mL/min with an increasing gradient of mobile phase B from 0 to 100% in 39 min, finally, the column was washed by 100% mobile phase A for 1 min. In total, 18 fractions were collected and lyophilized for phosphopeptide enrichment.

All fractions were subjected to phosphopeptide enrichment based on TiO_2_ beads. Samples were resuspended in 400 μL loading buffer (3% trifluoroacetic Acid (TFA)/70% ACN) and saturated by glutamic acid to close non-specific chemical groups. TiO_2_ was added to peptide mixture at a 1:10 peptide to TiO_2_ ratio (volume/weight) and reacted for 1 h at room temperature. To remove non-specific binding groups, the pellet was washed with wash buffer 1 (0.5% TFA/50% ACN) and wash buffer 2 (0.1% TFA/50% ACN), successively. After washing three times, phosphopeptides in the pellet were eluted by 100 μL NH_4_OH and then vacuum freeze-dried. Before MS analysis, the phosphopeptides were dissolved in 20 µL 0.1% formic acid.

### 5.4. Proteomics Profiling Based on LC-MS

Total protein samples were analyzed using the reverse phase Eksigent nanoLC ultra and ChiPLC-nanoflex (Eksigent, Redwood, CA, USA) combined with an AB SCIEX Triple TOF 5600 System (AB SCIEX, Concord, ON, Canada). The peptide digests were resolved in 0.1% formic acid and injected into ChromXP C18 (3 µm, 120 Å) nanoLC trap column. Peptides were eluted from an analytical column (15 cm, ID 75 µm, 3 µm, C18) at a flow rate of 300 mL/min using a linear gradient of 5–35% solvent B (99.9% acetonitrile with 0.1% formic acid) over 80 min. The mass spectrometer was operated with full scans in 350–1500 *m/z* range, and data acquired in the TOF mass analyzer with a resolution of 100,000 at 400 *m/z*, followed by MS/MS on the 28 most intense precursor ions from a survey scan with dynamic exclusion setting: a repeat count of 1 and exclusion duration of 18 s. Protein identification and quantification was performed with ProteinPilot (version 4.0, Sciex Inc, Framingham, MA, USA). The search parameters included: carbamidomethylation of cysteine as a fixed modification, oxidation of methionine, N-terminal acetylation and phosphorylation at serine, threonine and tyrosine as variable modifications. Precursor mass tolerance was set at 20 ppm and fragment mass tolerance of 0.05 Da. Enzyme specificity was set to trypsin and a maximum of two missed cleavages were allowed.

### 5.5. Data Quality Control and DEPs Identification

In order to ensure the availability of data, it is necessary to control the quality of the original data. Firstly, for peptides, the missed cleavages should be removed, and the peptides automatically annotated with a score of 1 should be retained. For proteins, the phosphorylated proteins with Conf ≥ 95% were selected. Second, the deletion of peptides and proteins with missing values caused by the machine. Then, the redundancy was removed, leaving only the unique peptides and proteins. Differentially expressed phosphorylated proteins and peptides between case and control samples were identified using a combination of Student’s *t*-test and fold-change (FC) for significance analysis. The criterion of statistical significance was *p*-value < 0.05, |log2(foldchange)| > 0.263.

### 5.6. Identification of Overrepresented Kinase Targeting Peptide Motifs

In order to obtain the types of overrepresented kinases that were activated after treated with spider toxin HNTX-III, we analyzed the amino acid sequences that tightly surround the phosphorylation sites of the 1779 identified non-redundant phosphopeptides by Motif-X online research based on the IPI *Rat* proteome database [55]. This program was conducted with central character of S/T/Y, motif width of 13, occurrences threshold of 20 and significance set at 1.00 × 10^−6^.

### 5.7. Functional Annotation and Pathway Analysis

To sufficiently decipher the biological functions of the differentially expressed phosphoproteins, we annotated them using the DAVID online database and tool [56]. DAVID is a powerful database dedicated to data annotation, visualization, and integration. All the corresponding gene symbols of phosphorylated differentially expressed proteins were imported into the database for gene functional annotation and enrichment analysis. Significant terms generated from the above analysis were visualized using R package ggplot2 *p*-value < 0.05 was set as the threshold for statistical significance.

### 5.8. PPI and Network Analysis

Protein-protein interaction network is helpful to dig out many databases of protein interactions of core regulatory genes, and STRING database is definitely the one that covers the most species and has the largest interaction information [57]. The DEPs identified in the experimental group I and II were imported into the STRING database to obtain the protein-protein interaction relationship, and then the visualization function of the combined analysis software Cytoscape was used to construct a PPI network [58].

### 5.9. Gene-Disease Associations Study

The DisGeNET database is a database of gene and mutation sites related to human diseases [59]. The platform also proposes a plug-in that can be run through Cytoscape software 7.3.0. Through the database, you can learn about the types of diseases associated with the genes of interest. The resulting data can then be visualized in the form of a network diagram using the DisGeNET plug-in in Cytoscape.

## Figures and Tables

**Table 1:** Three specific DEPs reversely regulated after HNTX-III and JZTX-I treatment.

Official Symbol	Official Full Name	*p*-Value ^1^		Fold Change ^2^	
		114:116 ^3^	115:116 ^4^	114:116	115:116
U2AF2	U2 snRNP auxiliary factor large subunit	1.18 × 10^−2^	1.72 × 10^−2^	0.43	1.61
EEF2	Elongation factor 2	2.12 × 10^−2^	9.79 × 10^−3^	0.63	4.13
FLNC	Filamin-C	1.75 × 10^−2^	9.34 × 10^−3^	0.64	2.17

^1^*p*-value < 0.05 is defined as a significant difference between the experimental group and the control group. The values here are presented in scientific notation. ^2^ Fold change value refers to the ratio of the expression level of the experimental group and the control group. Here it is defined as >1.20 is up-regulation, and <0.83 is down-regulation. ^3^ The HNTX-III treatment (114 iTRAQ tags) with blank group (116 iTRAQ tags) named as experimental group I. ^4^ The JZTX-I treatment (115 iTRAQ tags) with the blank group (116 iTRAQ tags) named as experimental group II.

## Data Availability

Data is contained within the article or Supplementary Materials.

## Supplementary Materials

The following are available online at https://www.mdpi.com/article/10.3390/toxins13080554/s1, Table S1: 436, 436, and 408 phosphorylated proteins identified in the replicate 1, replicate 2 and replicate 3, respectively; Table S2: 1144, 1182 and 1126 phosphorylated peptides were identified in the replicate 1, replicate 2 and replicate 3, respectively.
